# Higher adjuvant radioactive iodine therapy dosage helps intermediate-risk papillary thyroid carcinoma patients achieve better therapeutic effect

**DOI:** 10.3389/fendo.2023.1307325

**Published:** 2024-01-17

**Authors:** Xue Li, Hongyuan Zheng, Chao Ma, Yanhui Ji, Xuan Wang, Danyang Sun, Zhaowei Meng, Wei Zheng

**Affiliations:** ^1^Department of Nuclear Medicine, Tianjin Medical University General Hospital, Tianjin, China; ^2^Department of Neurology, Tianjin Beichen Traditional Chinese Medicine Hospital, Tianjin, China; ^3^Department of Nuclear Medicine, Tianjin Medical University General Hospital Airport Hospital, Tianjin, China

**Keywords:** intermediate-risk papillary thyroid carcinoma, adjuvant radioactive iodine therapy, dosages, therapy response, decision curve analysis (DCA)

## Abstract

**Objective:**

This retrospective study aims to evaluate the therapeutic effect of varying dosages of adjuvant radioactive iodine (RAI) therapy on intermediate-risk papillary thyroid carcinoma (PTC) patients.

**Methods:**

This retrospective study involved a total of 427 intermediate-risk PTC patients, out of which 202 received a 3.7GBq dosage of RAI, and 225 received a 5.55GBq dosage. The evaluation involved assessing the therapeutic outcomes, number of treatment cycles, and successful remnant ablation rates in both dose groups, six months post-adjuvant RAI therapy. Univariate and multivariate logistic regression analyses were employed to identify factors linked with excellent response (ER). Following this, prognostic nomograms were constructed to provide a visual representation of the prediction models. Calibration curves, the concordance index (C-index), and the receiver operating characteristic (ROC) curve were employed to evaluate the predictive performance of these nomograms. The Hosmer-Lemeshow test was applied to assess the models’ goodness-of-fit. Additionally, the clinical utility of the prognostic nomograms was appraised through decision curve analysis (DCA)

**Results:**

The high-dose (HD) group exhibited significantly higher proportions of ER, single treatment cycles, and successful remnant ablation rates (p<0.05). Being male, receiving a 3.7GBq dose, having an N1b stage, an sTg level ≥10ng/ml, or an sTg/TSH ratio ≥0.11 were independent risk factors for Non-ER. Two prognostic nomograms, “sTg Nomogram” and “sTg/TSH Nomogram”, were established. The ranking of factors contributing to ER, in descending order, included the sTg or sTg/TSH ratio, N stage, therapy dosage, sex, and soft tissue invasion. The “sTg/TSH Nomogram” demonstrated a higher C-index compared to the “sTg Nomogram”. The calibration curves indicated excellent calibration for both nomograms. DCA demonstrated that the net benefit of the “sTg/TSH Nomogram” was higher than that of the “sTg Nomogram”.

**Conclusion:**

Higher initial RAI therapy doses can improve therapeutic efficacy for intermediate-risk PTC patients. The developed nomograms, particularly the “sTg/TSH Nomogram”, could assist clinicians in optimal therapeutic decision-making.

## Introduction

Differentiated thyroid cancer (DTC), the most common endocrine malignancy, constitutes 90% of all thyroid cancers. The primary treatment modalities for DTC include surgery, postoperative radioactive iodine (RAI) therapy, and thyrotropin (TSH) suppression. The ATA classifies DTC patients into low, intermediate, and high-risk groups based on recurrence and response to therapy. Depending on the postoperative risk stratification of the individual patients, the primary goal of RAI after total thyroidectomy includes RAI remnant ablation, RAI adjuvant therapy and RAI therapy (1). RAI therapy is routinely recommended for patients with high-risk DTC. Moreover, adjuvant RAI therapy is selectively employed for intermediate-risk patients to control persistent disease and prevent recurrence. Although these patients typically have good long-term prognoses due to the lack of distant metastases, appropriate surgical resection, and adjuvant RAI therapy, the frequency of persistent disease remains high (2). Adjuvant RAI therapy can benefit intermediate-risk DTC patients with a relatively high risk of recurrence by destroying suspected but unconfirmed residual disease (3, 4). Some studies have shown that RAI can improve overall survival or disease-specific death of intermediate-risk DTC patients (5, 6). However, consensus on the appropriate RAI dosage for adjuvant therapy in intermediate or high-risk patients is yet to be reached, particularly for those in the intermediate-risk category. Factors such as soft tissue invasion, extensive cervical lymph node metastasis, and relatively high serum thyroglobulin (Tg) levels might contribute to a higher risk of recurrence in intermediate-risk DTC patients. It remains unclear whether patients receiving a lower dosage can achieve a satisfactory response after initial RAI therapy and whether they might need additional RAI therapy due to an indeterminate response or biochemical incomplete response.

This study aims to evaluate the therapeutic impact of varying adjuvant RAI therapy dosages (3.7GBq and 5.5GBq) in intermediate-risk DTC patients, and to identify influencing factors for an excellent response (ER) to initial therapy. Prior to administering RAI, the post-operative Tg level plays a crucial role in both monitoring the disease and guiding management decisions (7–10) Notably, Tg levels can be influenced by thyroid-stimulating hormone (TSH) levels. Our previous research has indicated that the sTg/TSH ratio may serve as an additional predictor of the therapeutic effects of RAI (11). Consequently, we aim to develop two prognostic nomograms: the “sTg Nomogram” and the “sTg/TSH Nomogram, “ to predict the probability of ER to treatment. The discrimination, accuracy, calibration, and clinical utility of these two nomograms will be assessed using the concordance index (C-index), receiver operating characteristic (ROC) curve, calibration curve, and decision curve analysis (DCA).

## Materials and methods

### Patients

We conducted a retrospective review of the clinical records of patients who underwent total thyroidectomy and postoperative RAI therapy for papillary thyroid carcinoma (PTC) at the Department of Nuclear Medicine, Tianjin Medical University General Hospital, Tianjin, China, from March 2018 to July 2021. According to the ATA guidelines (version 2015), our study included patients with intermediate-risk PTC confirmed by surgical histopathology who received an RAI dose of either 3.7GBq or 5.55GBq. Patients with a post-operative Tg level highly suggestive of distant metastases or positive anti-thyroglobulin antibodies (TgAb) were excluded. Patients were divided into two groups: a Low-Dose (LD) group where 202 patients received 3.7GBq RAI therapy, and a High-Dose (HD) group where 225 patients received 5.55GBq RAI therapy.

### Postoperative radioiodine therapy and follow-up

Patients underwent RAI therapy after discontinuing levothyroxine (L-T4) for 2-4 weeks until TSH levels exceeded 30mIU/L, and adhered to a low-iodine diet for a minimum of 2 weeks. Prior to ^131^I administration, serum stimulated thyroglobulin (sTg), TgAb, and TSH levels were measured. Neck ultrasonography (US) and chest CT were performed simultaneously. Post-ablation whole body scans (WBS) were carried out 3 to 5 days after RAI therapy. Patients resumed L-T4 immediately after RAI therapy for TSH suppression. Thyroid hormones, TSH, Tg, TgAb were typically examined at 1-, 3-, and 6-months post RAI therapy, and neck US performed every 3 months. During this period, modulation of L-T4 dosage was undertaken as needed. A diagnostic ^131^I scan was performed 6 months post RAI therapy. Additional RAI therapy was initiated if there were ^131^I-avid recurrence or metastatic lesions, or rising sTg levels. In cases where clinical suspicion of recurrence and metastatic lesions existed, additional imaging studies, such as neck/chest CT or F-18-fluorodeoxyglucose positron emission tomography, were conducted.

### Study design and determination of clinical outcome

We reviewed all patients’ clinical records to collect data on sex, age at diagnosis, T stage, N stage, American Joint Cancer Control (AJCC) stage (8th edition), soft tissue invasion, pre-therapy TSH and sTg levels, and sTg/TSH ratio. Owing to the absence of comprehensive clinicopathological data in certain patients, some clinicopathological characteristics, including extrathyroidal extension, tumor focality, tumor laterality, and lymphovascular invasion, were not incorporated in our analysis. The therapy response to RAI was evaluated at 6 months post-RAI therapy. As per the ATA guideline (version 2015), the responses to therapy included ER (negative imaging and either sTg<1ng/mL or suppressed Tg <0.2ng/mL), Biochemical Incomplete Response (BIR) (negative imaging and suppressed Tg ≥1ng/mL or sTg ≥10ng/mL or rising TgAb levels), Structural Incomplete Response (SIR) (structural or functional evidence of disease with any Tg level with or without TgAb), and Indeterminate Response (IDR) (nonspecific findings on imaging studies, faint uptake in thyroid bed on RAI scanning, non-detectable sTg <1ng/mL, detectable sTg <10ng/mL or stable or declining TgAb in the absence of structural or functional disease) (1). BIR and SIR were collectively referred to as Incomplete Response (IR).

We compared the therapeutic effects and successful remnant ablation (where diagnostic WBS found no residual thyroid bed uptake regardless of sTg levels) rates of different dose groups in all patients at 6 months post-adjuvant RAI therapy. If ^131^I-avid recurrence or metastatic lesions were observed, or rising sTg levels were noted, patients underwent subsequent RAI therapy. Thus, we also compared the treatment cycles across different dose groups.

We then conducted univariate and multivariate logistic regression analyses to explore the influence of various factors such as therapy dose, sex, age at diagnosis, T stage, N stage, AJCC stage, soft tissue invasion, pre-therapy TSH, sTg level, and sTg/TSH ratio on the ER probability. Given the collinearity between sTg and sTg/TSH ratio, we conducted multivariate logistic regression analysis, including sTg or sTg/TSH ratio with other variables, respectively.

Finally, we developed the prognostic nomograms “sTg nomogram” and “sTg/TSH nomogram” to predict the ER probability. The calibration curve, ROC curve and decision curve analysis (DCA) were utilized to evaluate the performance of the two prediction models.

### Statistical analysis

Continuous variables, with an abnormal distribution, are represented as the median along with interquartile ranges. Categorical variables are displayed as numbers with their corresponding percentages. Differences between continuous variables in the dose groups were evaluated using the Mann-Whitney test, while a Chi-squared test was utilized to compare categorical variables. Univariate and multivariate logistic regression were applied to assess predictors of ER. Initially, univariate analysis was performed, and any variable with a p-value <0.1 in this analysis was included in the multivariate regression. A p-value <0.05 was considered statistically significant.

The discrimination of these models was evaluated using the Concordance index (C-index). Bootstrap resampling with 1000 iterations was employed for the internal validation of the predictive capabilities of these two nomograms. The calibration curve was utilized to assess the accuracy of these nomograms in estimating outcomes and to check for overfitting. A curve closely aligning with the 45-degree line indicates good calibration of the prediction model. Additionally, the Hosmer-Lemeshow test was conducted to assess the goodness-of-fit of the models. This test compares the expected event numbers from the prediction model with the observed event numbers. A p-value > 0.05 suggests a well-fitted model. The ROC curve was drawn to determine the accuracy of the prediction models. The area under the ROC curve (AUC) corresponds to the C-index in binary logistic regression analysis. DCA was used to evaluate the clinical utility of the predictive nomograms. In DCA, the x-axis represents the threshold probability for an ER, and the y-axis denotes the net benefit. The “sTg Nomogram” and “sTg/TSH Nomogram” were compared against scenarios where all or none of the patients have an ER. The nomogram demonstrating a higher net benefit is considered to have greater clinical utility.

R software (version 4.0.5, http://www.r-project.org/) was employed to perform the nomogram, Hosmer-Lemeshow test, calculate C-index, draw calibration curve, ROC curve and DCA. All other statistical analyses were conducted using SPSS (version 24.0; SPSS Inc., Chicago, Illinois, USA). The study received approval from the Institutional Review Board of the Tianjin Medical University General Hospital.

## Results

No significant differences in clinical and pathologic characteristics were observed between the two groups. (Refer to **Table 1**).

**Table 1:** Clinical and pathologic characteristics at baseline.

Characteristics	LD (n=202)	HD (n=225)	P
Age at diagnosis	42(34-49.25)	42(34-50.50)	0.799
Sex Male Female	69(34.2%) 133(65.8%)	86(38.2%) 139(61.8%)	0.383
T stage 1 2 3	168(83.2%) 20(9.9%) 14(6.9%)	167(74.2%) 29(12.9%) 29(12.9%)	0.059
N stage N0 1a 1b	7(3.5%) 108(53.5%) 87(43.1%)	3(1.3%) 105(46.7%) 117(52.0%)	0.089
AJCC stage I II	169(83.7%) 33(16.3%)	187(83.1%) 38(16.9%)	0.878
Soft tissue invasion No Yes	151(74.8%) 51(29.1%)	155(68.9%) 70(31.1%)	0.179
Pre-therapy TSH(*u*IU/mL)	76.665(54.817-100.000)	70.332(51.948-96.285)	0.080
sTg(ng/mL)	2.46(0.94-6.79)	2.41(0.70-6.21)	0.582
sTg/TSH	0.038(0.014-0.095)	0.036(0.011-0.100)	0.894

Dates are expressed as the median (percentiles 25-75) or frequencies.

LD, low-dose, 3.7GBq; HD, high-dose, 5.55GBq.

### Comparison of responses to initial RAI, treatment cycles, and successful remnant ablation rates between dose groups

As illustrated in **Table 2**, we identified a significant difference in the proportions of ER and Non-ER between the two groups. The HD group exhibited a significantly higher ER rate than the LD group (57.3% *vs* 44.1%, p<0.05). Although the proportions of BIR, SIR, and IDR in the HD group were lower than those in the LD group, these differences were not statistically significant (6.2% *vs* 10.4%, 5.3% *vs* 5.9%, 31.1% *vs* 39.6%, p>0.05, respectively). The HD group had significantly higher proportions of single treatment cycle (84.0% *vs* 75.7%, p<0.05) and a higher successful remnant ablation rate (92.4% *vs* 83.7%, p<0.05).

**Table 2:** Responses to initial RAI, treatment cycles and successful remnant ablation rates.

Characteristics	LD (n=202) N (%)	HD (n=225) N (%)	P
Response			
ER	89(44.1)	129(57.3)	0.042*
BIR	21(10.4)	14(6.2)	
SIR	12(5.9)	12(5.3)	
IDR	80(39.6)	70(31.1)	
ERNon-ER	89(44.1)113(55.9)	129(57.3)96(42.7)	0.006*
BIR Non-BIR	21(10.4) 181(89.6)	14(6.2) 211(93.8)	0.116
SIR Non-SIR	12(5.9) 190(94.1)	12(5.3) 213(94.7)	0.786
IDR Non-IDR	80(39.6) 122(60.4)	70(31.1) 155(68.9)	0.066
Treatment cycles Single Multiple	153(75.7) 49(24.3)	189(84.0) 36(16.0)	0.033*
successful remnant ablation Yes No	169(83.7) 33(16.3)	208(92.4) 17(7.6)	0.005*

LD, low-dose, 3.7GBq; HD, high-dose, 5.55GBq.

ER, excellent response; BIR, biochemical incomplete response; SIR, structural incomplete response; IDR, indeterminate response; IR, incomplete response.

*p<0.05.

### Univariate and multivariate logistic regression analysis for potential factors of ER to RAI

**Table 3** shows that univariate logistic regression analysis found no significant association between therapeutic effects and age at diagnosis, T stage, AJCC stage, and pre-therapy TSH levels. However, sex, therapy dose, N stage, soft tissue invasion, sTg level, and sTg/TSH ratio could potentially affect the therapeutic effects (P<0.1). These factors were further evaluated using multivariate logistic regression analysis to assess their predictive value. We addressed the collinearity between sTg and the sTg/TSH ratio by conducting separate multivariate logistic regression analyses for each. The results were nearly identical, leading us to present only the sTg prediction model data in **Table 3**. However, the OR value and 95%CI of the sTg/TSH ratio are also provided. Results revealed that being male, receiving a 3.7GBq dose, having an N1b stage, an sTg level ≥10ng/ml, and an sTg/TSH ratio ≥0.11 were independent risk factors for Non-ER.

**Table 3:** Univariate and multivariate logistic regression analysis based on all variables for ER.

Variables	Univariate analysis		Multivariate analysis	
	OR (95%CI)	P value	OR (95%CI)	P value
Age at diagnosis <45 45≤Age<55 ≥55	Reference 1.004(0.633-1.594) 1.454(0.863-2.450)	0.986 0.160		
Sex Male Female	Reference 1.639(1.101-2.440)	0.015	Reference 1.653(1.050-2.603)	0.030*
Therapy dose 3.7GBq 5.55GBq	Reference 1.706(1.163-2.503)	0.006	Reference 2.464(1.571-3.866)	<0.001*
T stage 1 2 3	Reference 0.781(0.428-1.427) 1.332(0.701-2.533)	0.422 0.382		
N stage N0 1a 1b	Reference 0.311(0.064-1.497) 0.205(0.043-0.991)	0.145 0.049	Reference 0.268(0.043-1.653) 0.159(0.026-0.986)	0.156 0.048*
AJCC stage I II	Reference 1.481(0.883-2.483)	0.136		
Soft tissue invasion No Yes	Reference 0.698(0.457-1.065)	0.096	Reference 0.732(0.442-1.212)	0.225
Pre-therapy TSH(*u*IU/mL) 30≤TSH<60 60≤TSH<90 TSH≥90	Reference 0.716(0.446-1.149) 1.105(0.696-1.754)	0.166 0.672		
Stimulated Tg(ng/mL) <10 ≥10	Reference 0.057(0.022-0.145)	<0.001	Reference 0.054(0.021-0.141)	<0.001*
sTg/TSH <0.11 ≥0.11	Reference 0.074(0.037-0.149)	<0.001	Reference 0.066 (0.032-0.136)	<0.001*

*p<0.05.

### Prognostic nomogram for ER probability

**Figure 1:**
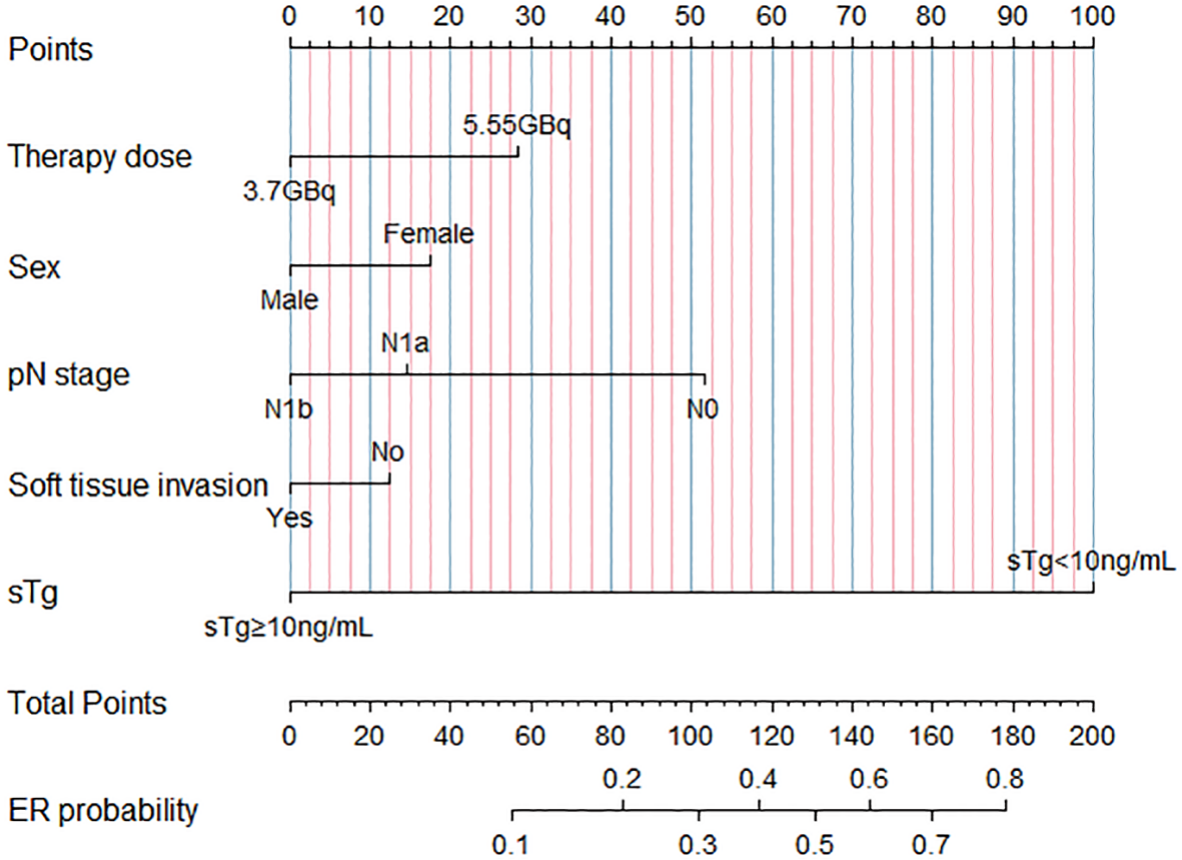
“sTg nomogram” predicting ER rates for intermediate-risk PTC patients receiving RAI.

**Figure 2:**
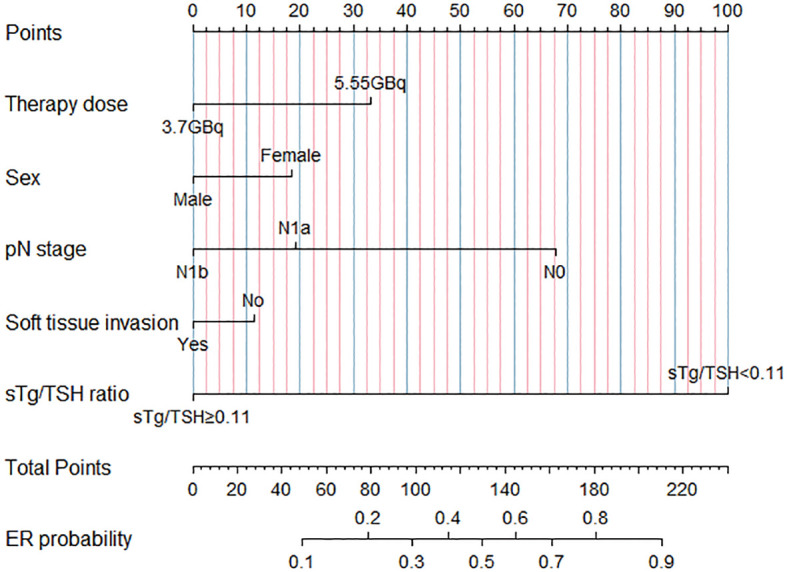
“sTg/TSH nomogram” predicting ER probability for intermediate-risk PTC patients receiving RAI.

**Figure 3:**
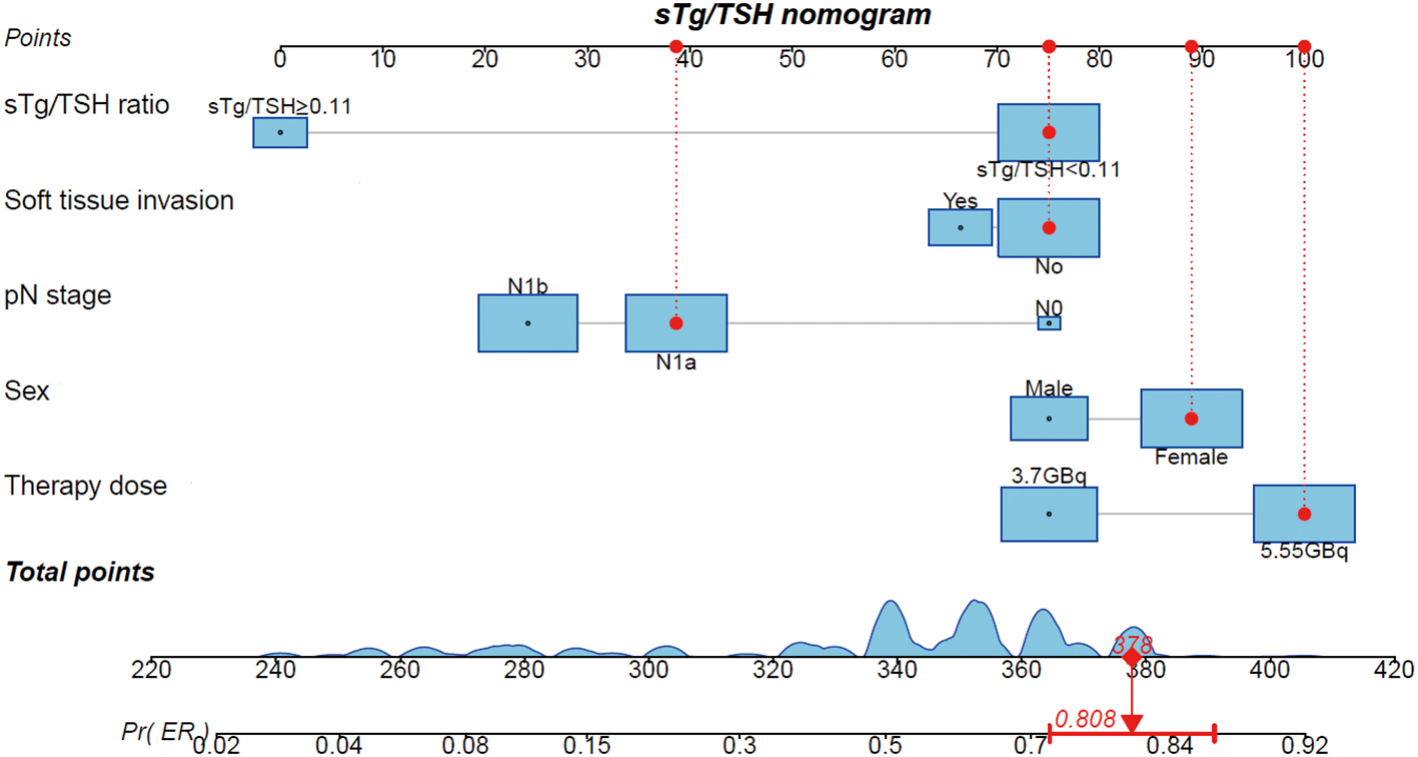
Individual “sTg/TSH nomogram” predicting ER probability for a female intermediate-risk PTC patient with N1a, no soft tissue invasion, sTg/TSH<0.11 receiving 5.55GBq RAI therapy.

Using the results from logistic regression analysis, we developed two prognostic nomograms, “sTg nomogram” and “sTg/TSH nomogram”, to predict ER probability (**Figures 1**, **2**). A score was assigned to each variable by drawing a perpendicular line from the corresponding axis to the top line labeled “POINTS”. The scores of all variables were then added, and a line was drawn from the axis labeled “Total Points” to estimate the ER probability. From the nomogram, we found that sTg or sTg/TSH ratio, N stage, and therapy dose were the top three contributors to ER, followed by sex and soft tissue invasion. To make the nomogram more illustrative, we developed an individual “sTg/TSH nomogram” for a female patient with N1a, no soft tissue invasion, sTg/TSH<0.11, who received 5.55GBq RAI therapy. The total points, derived from adding all variable scores, was 378, corresponding to an ER probability of 80.8% (**Figure 3**).

### Internal validation and clinical usefulness of “sTg nomogram” and “sTg/TSH nomogram”

**Figure 4:**
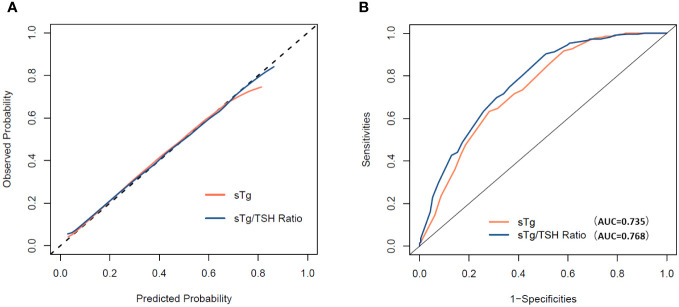
**(A)** Calibration curve for the “sTg nomogram” and “sTg/TSH nomogram”. **(B)** ROC curve for the “sTg nomogram” and “sTg/TSH nomogram”.

The C-index of “sTg/TSH nomogram” was 0.768 (95% CI: 0.724 to 0.813), which was better than that of the “sTg nomogram” 0.735 (95% CI: 0.688 to 0.783). Hosmer-Lemeshow test shown that two prediction models were well fitted. Chi-squared of “sTg nomogram” and “sTg/TSH nomogram” were 3.130 and 1.740, p=0.926 and 0.988 respectively. The calibration curve was shown in **Figure 4A**, both the two nomograms had good calibration for predicting the ER probability. ROC curve was shown in **Figure 4B**, the AUC of the two nomograms were same with C-index, the AUC of “sTg/TSH nomogram” is slightly higher than that of “sTg nomogram.

**Figure 5:**
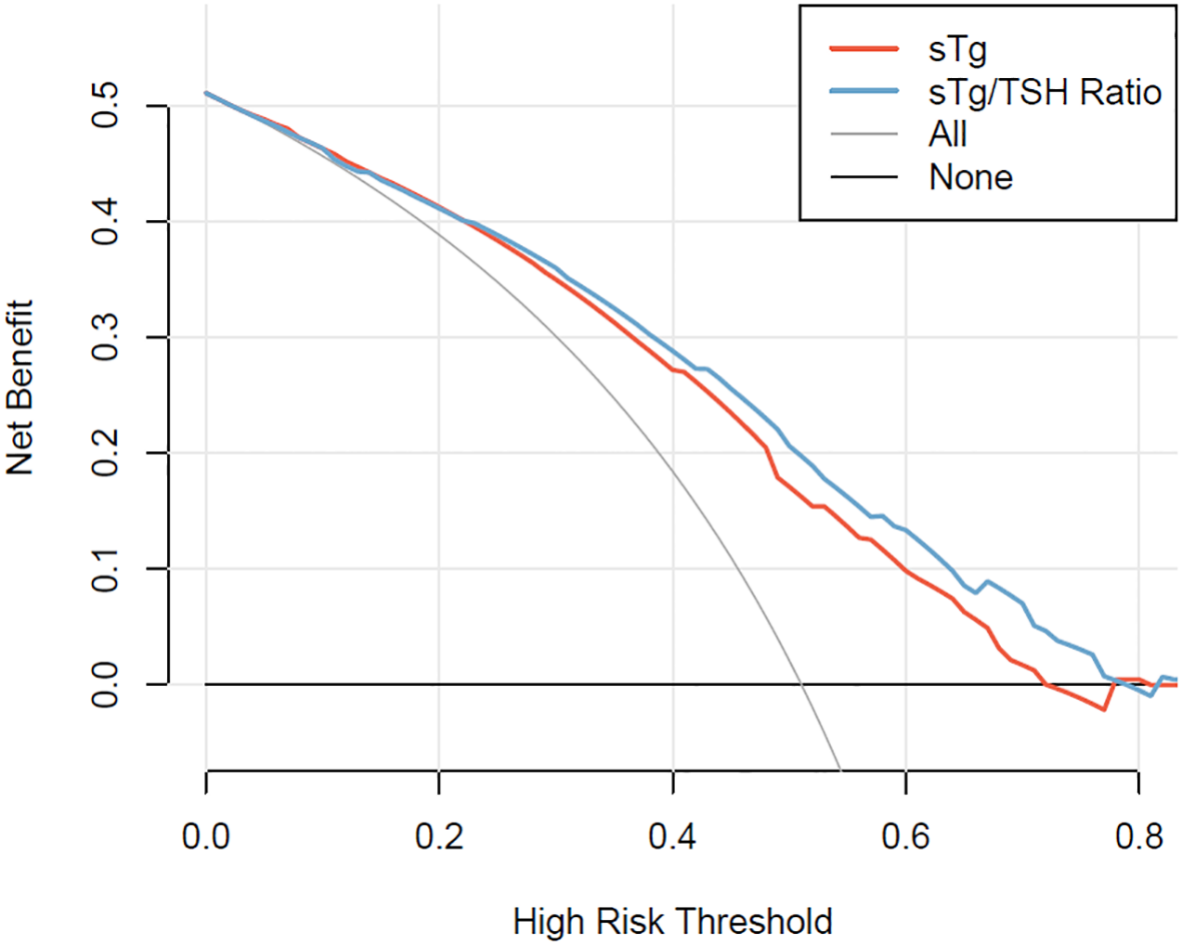
Decision curves of the “sTg nomogram” and “sTg/TSH nomogram”.

DCA for the “sTg nomogram” and “sTg/TSH nomogram” is presented in **Figure 5**. Both the “sTg nomogram” and “sTg/TSH nomogram” demonstrated greater positive net benefits across a wide range of ER threshold probabilities compared to scenarios where all or no patients achieved ER. This indicates their favorable clinical usefulness in predicting ER probability. When compared to the “sTg nomogram”, the net benefits of the “sTg/TSH nomogram” were slightly higher.

## Discussion

Differentiated thyroid carcinoma (DTC), being a slow-growing malignancy, often grants patients an excellent long-term prognosis when carefully managed. Clinical observations reveal that a significant number of intermediate-risk DTC patients display persistent small-volume structural disease or abnormal postoperative thyroglobulin values, indicating the persistence of disease (12). Studies suggest that adjuvant therapy can have beneficial effects in up to 30% of DTC patients by destroying potentially malignant cells or occult multifocal disease (13). Research conducted by Ruel et al. established a positive correlation between radioactive iodine (RAI) treatment and increased overall survival, noting a 29% reduced risk of death when RAI was administered in the postoperative setting (5). In a similar vein, a study by Al-Qahtani et al. presented evidence that adjuvant 131I ablation improves disease-free survival rates in papillary microcarcinoma patients, especially those with poor prognostic factors (14). Before initiating RAI therapy, it poses a significant challenge to distinguish structural or functional disease from thyroid remnants or biochemical disease based on serum thyroglobulin (sTg) levels. A study demonstrated 82.7% DTC patients with unexplained hyperthyroglobulinemia were finally attributed to biochemical, functional, or structural disease, and 81.5% of these patients yielded a non-structural or functional incomplete response 6-12 months post 5.55 GBq adjuvant RAI therapy (15). This result suggests that 5.55GBq of RAI can effectively identify and treat the lesions, especially when the overall tumor burden is relatively low (15). Despite these findings, the literature lacks sufficient information to provide guidance on the optimal administered activity for patients undergoing adjuvant therapy (12). The benefits of large RAI dosages remain uncertain to this date (16). Accordingly, the American Thyroid Association (ATA) guidelines have adjusted the dosage recommendations for adjuvant therapy, reducing the range from 3.7-7.4 GBq to 1.11-5.55 GBq (1).

In certain past studies, the therapeutic efficacy of intermediate-risk DTC patients treated with higher doses of RAI therapy (3.7GBq or 5.55GBq) has not been found to be superior to those treated with lower doses (1.11GBq or 3.7GBq) (17–21). Moreover, 3.7GBq RAI therapy is often associated with a higher frequency of short-term adverse effects compared to 1.11GBq or 1.875GBq (17, 20, 21). Nevertheless, another report indicated that 5.55GBq RAI is associated with a lower recurrence rate than 3.7 GBq RAI for high-risk DTC patients (22). In the study conducted by Jeong et al., when the diagnostic whole-body scans (WBS) findings of residual thyroid bed uptake were not considered as criteria for ablation success and reliance was placed only on sTg and/or neck US, no significant difference in the ablation success rate was found between 3.7 GBq and 5.55GBq. However, when the diagnostic WBS findings were included as the assessment index, the ablation success rate of 3.7GBq was significantly lower compared to 5.55GBq (18). It is important to note that these studies involved patients with diverse clinicopathological features, recurrence risks, and RAI dosages. The subjects in our study were intermediate-risk PTC patients. We evaluated their responses to LD or HD RAI therapy six months post-initial RAI therapy. According to the 2015 ATA guidelines, the responses to initial surgery and RAI therapy were dynamically evaluated based on Tg levels and follow-up diagnostic image results. Our observations revealed differences in response to RAI therapy between the LD and HD groups. A significant difference was noted primarily in the ER rate, with the HD group exhibiting a higher ER rate than the LD group (57.3% *vs* 44.1%, p<0.05). Furthermore, the proportions of IR, BIR, SIR, IDR in HD group were lower than that in LD group, although these differences did not reach statistical significance. In 2017, a study reported that patients in the LD group demonstrated higher rates of BIR or SIR (3). Similar to our study, all patients in that research were intermediate-risk DTC patients. However, the RAI dosage in the LD group was 1.11GBq, and the patients in the HD group received 3.7GBq or 5.55GBq of RAI. The study revealed that 28.75% of patients in the LD group and 11.29% in the HD group exhibited remnant thyroid uptake. Another study suggested that insufficient RAI dosage could be one of the likely causes for frequent observations of residual thyroid bed uptake, and higher RAI dosages are more beneficial for the ablation of large normal-thyroid remnants (18). For intermediate-risk PTC patients, our disease management team advises an adjuvant RAI therapy dosage ranging from 3.7 to 5.55 GBq, depending on the clinicopathological features. Additionally, we observed that remnant thyroid uptake was more frequently noted in the LD group than the HD group six months post-RAI therapy (16.3% *vs* 7.6%, p<0.05).

A previous study revealed that the number of treatment cycles and the accumulated RAI dose had differential impacts on the complete blood count of patients of different sexes and ages (23). In our comparison of treatment cycles between different dose groups, we found that the HD group had significantly higher proportions of one-time treatments. This indicates that the accumulated RAI dose of some patients in the HD group is less than those in the LD group. This result could lead to cost savings and reduced adverse effects in some patients, such as transient neck pain and edema, salivary dysfunction, nasolacrimal obstruction, and hematological toxicity.

It is well understood that certain tumor clinicopathological features have a profound impact on prognosis. The role of initial prognostic parameters is fundamental in guiding management recommendations. Undeniably, adequate treatment is essential for achieving complete or partial remission (24). In the literature, several studies have found that the size, number, and ratio of metastatic lymph nodes and extranodal extension indicate an unfavorable clinical outcome (25–27). Serum postoperative sTg and TgAb levels, which are sensitive and specific biomarkers for DTC, form part of the early postoperative disease status evaluation and impact clinical decision-making (16). Therefore, the determination of RAI dosage should take clinicopathological features into full consideration. In our study, we performed univariate and multivariate logistic regression to evaluate the predictors of ER. According to ATA guidelines, the optimal cut-off value of postoperative sTg level to guide decision-making regarding RAI administration is not known (1). A previous study by our team suggested that a pre-^131^I treatment sTg level of less than 9.5ng/mL predicted a better therapeutic effect (11). A meta-analysis involving 3947 patients by Webb et al. demonstrated that postoperative sTg levels of less than 10ng/mL indicate a better prognosis with adjuvant RAI therapy (7). Similarly, Piccardo et al. confirmed that an sTg level of less than 10ng/mL is a strong predictor of complete remission after initial treatment, with a very high negative predictive value (93%) (24). Hence, we considered 10ng/mL as the cut-off value of sTg level for logistic regression analysis. It is widely known that sTg levels can be influenced by TSH levels. Therefore, in this study, we used the sTg/TSH ratio as another predictor of RAI therapeutic effects, using TSH to correct the predictive value of sTg. Given the collinearity between sTg and sTg/TSH, we analyzed sTg and sTg/TSH separately as influencing factors. A previous study by our team confirmed that an sTg/TSH ratio of less than 0.11 before the first RAI therapy predicted a better therapeutic effect (11). In the present study, we maintained 0.11 as the cut-off value for the sTg/TSH ratio. All the patients in our study were intermediate-risk PTC, and were divided into stages I and II by the age of 55 years according to the 8th edition of the ATCC TNM classification system. The 7th edition of the AJCC TNM classification system used the age of 45 years as a cut-off to upstage patients. Our previous study also showed that papillary thyroid microcarcinomas in patients aged under 45 years were more aggressive, especially regarding lymph node metastasis (28). Therefore, in logistic regression analysis, we set the ages of 45 and 55 years as the cut-off points to distinguish the AJCC stage.

Univariate and multivariate logistic regression analyses in our study confirmed that therapy dose is an independent risk factor for an ER. The ER rate of patients in the HD group was 2.464 times higher than those receiving LD RAI therapy. Furthermore, both sTg and the sTg/TSH ratio showed a strong association with ER. Specifically, sTg ≥ 10ng/mL, sTg/TSH ≥ 0.11, and N1b might predict lower ER rates in intermediate-risk PTC patients receiving adjuvant RAI therapy. Interestingly, our study found male gender to be a risk factor for a non-ER. Previous studies have shown mixed results regarding whether gender impacts the efficacy of RAI treatment. However, most studies lean towards the conclusion that male gender is a risk factor for a poorer prognosis of RAI treatment (28–30), which is consistent with the results of our study. Additionally, we found no significant relationship between soft tissue invasion and ER. This result might be attributed to our research population, as we focused only on intermediate-risk PTC extending into the sternothyroid muscle or perithyroidal soft tissues, and not high-risk PTC extending beyond the capsule to invade subcutaneous soft tissues, larynx, trachea, esophagus, or the recurrent laryngeal nerve.

To better predict the therapeutic effects of initial RAI treatment in intermediate PTC patients, we constructed two nomograms - the “sTg nomogram” and the “sTg/TSH nomogram” - to predict ER probability based on logistic regression analysis. The factors contributing to ER, in descending order of impact, are sTg or sTg/TSH ratio, N stage, therapy dose, sex, and soft tissue invasion. These findings align with the results of our multivariate logistic regression analysis. In our study, the C-index and the AUC of these two nomograms were 0.735 and 0.768, respectively. This indicates that both nomograms demonstrated good discrimination ability and accuracy. Moreover, the “sTg/TSH Nomogram” outperformed the “sTg Nomogram” in terms of AUC and C-index. The calibration curves of both nomograms closely approached the ideal line, signifying excellent calibration. Additionally, the Hosmer-Lemeshow test results confirmed that both prediction models were well-fitted. Overall, these findings suggest that our prediction models exhibit exceptional discrimination, accuracy, and calibration. DCA is a tool that measures the net benefit of a diagnostic method or predictive model by jointly considering benefits and harms (31). DCA takes into account the clinical usefulness of a prediction model and guides clinical decision-making. Previous high-quality studies have used DCA to assess the net benefits of predictive models (32, 33). However, fewer studies have used DCA to evaluate predictive models for thyroid cancer. In our study, we calculated the net benefits of the “sTg nomogram” and “sTg/TSH nomogram”. The results of the DCA suggested that both the “sTg nomogram” and “sTg/TSH nomogram” have clinical utility, with the “sTg/TSH nomogram” providing slightly higher net benefits, indicating superior clinical usefulness.

Our study does have some limitations. First, being a retrospective study, we did not estimate the short-term adverse effects of the two different dose groups. Second, due to a lack of long-term follow-up, we analyzed only initial therapy responses and not recurrence-free survival rates or recurrence rates. Lastly, certain other clinicopathological features, such as characteristics of metastatic lymph nodes, BRAFV600E mutation, multifocality, and so on, were not included in this study. Considering these limitations, further investigations are warranted.

## Conclusion

In conclusion, different RAI doses have different therapeutic effects depending on patients’ clinicopathological features. A higher initial RAI dose can help increase the probability of an ER, improve the success rate of remnant ablation, and reduce the number of treatment cycles for intermediate-risk PTC patients. Independent risk factors for Non-ER include sTg/TSH≥0.11, sTg ≥10ng/mL, N1b stage, and male gender. For intermediate-risk PTC patients with these risk factors, a higher initial RAI dose should be considered. We have developed clinically useful nomograms for calculating ER probabilities in intermediate-risk PTC patients after initial RAI therapy. The prediction models with excellent discrimination, accuracy and calibration. The “sTg/TSH nomogram” performed better than the “sTg nomogram” and could assist clinicians in making optimal clinical decisions.

## Data availability statement

The raw data supporting the conclusions of this article will be made available by the authors, without undue reservation.

## Ethics statement

The studies involving humans were approved by Ethical Committee Tianjin Medical University General Hospital Tianjin, China. The studies were conducted in accordance with the local legislation and institutional requirements. Written informed consent for participation was not required from the participants or the participants’ legal guardians/next of kin in accordance with the national legislation and institutional requirements.

## Author contributions

XL: Data curation, Methodology, Writing – original draft, Writing – review & editing. HZ: Data curation, Writing – original draft. CM: Methodology, Writing – original draft. YJ: Methodology, Software, Writing – original draft. XW: Software, Writing – review & editing. DS: Data curation, Writing – review & editing. ZM: Methodology, Writing – original draft, Writing – review & editing. WZ: Methodology, Project administration, Writing – original draft, Writing – review & editing.
